# Chiral Bis(tetrathiafulvalene)-1,2-cyclohexane-diamides

**DOI:** 10.3390/molecules27206926

**Published:** 2022-10-15

**Authors:** Alexandra Bogdan, Ionuț-Tudor Moraru, Pascale Auban-Senzier, Ion Grosu, Flavia Pop, Narcis Avarvari

**Affiliations:** 1 Univ. Angers, CNRS, MOLTECH-Anjou, SFR MATRIX, F-49000 Angers, France; 2 Faculty of Chemistry and Chemical Engineering, Department of Chemistry, Babes-Bolyai University, 11 Arany Janos Str., 400028 Cluj-Napoca, Romania; 3 Laboratoire de Physique des Solides, Université Paris-Saclay CNRS UMR 8502, Bât. 510, F-91405 Orsay, France

**Keywords:** chirality, tetrathiafulvalene, chiroptical properties, crystal structure determination, DFT calculations, magnetic properties

## Abstract

Chiral bis(TTF) diamides have been obtained in good yields (54–74%) from 1,2-cyclohexane-diamine and the corresponding TTF acyl chlorides. The (*R*,*R*)-**1** and (*S*,*S*)-**1** enantiomers have been characterized by circular dichroism and the racemic form by single-crystal X-ray diffraction. The neutral racemic bis(TTF)-diamide shows the formation of a pincer-like framework in the solid state, thanks to the intramolecular S**···**S interactions. The chemical oxidation in a solution using FeCl_3_ provides stable oxidized species, while the electrocrystallization experiments provided radical cation salts. In particular, single-crystal resistivity measurements on the racemic donor with AsF_6_^−^ as a counterion demonstrate semiconductor behavior in this material. The DFT and TD-DFT calculations support the structural and chiroptical features of these new chiral TTF donors.

## 1. Introduction

**Figure 1:**
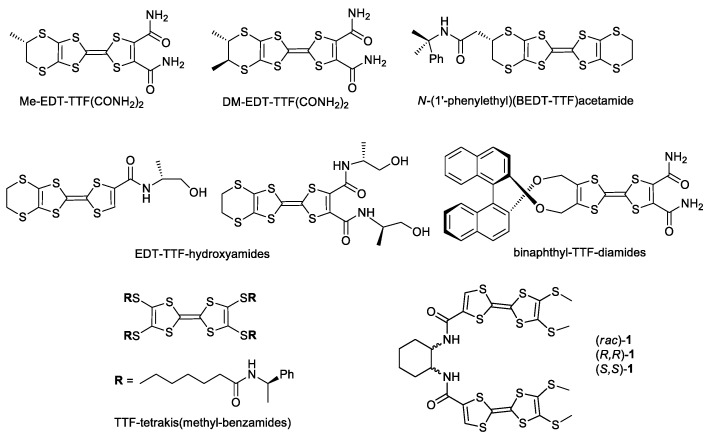
Examples of chiral TTF-amides; **1** is described in the present work.

Chiral tetrathiafulvalenes (TTF) [1] have aroused increasing interest since the reports of Wallis and Dunitz on the first enantiopure TTF, namely tetramethyl-bis(ethylenedithio)-TTF (TM-BEDT-TTF) [2], and some of its conducting radical cation salts [3]. In particular, the observation of the synergistic effect between chirality and conductivity under a magnetic field applied parallel to the direction of the current, referred to as the electrical magneto-chiral anisotropy (eMChA) effect [4,5], in the case of the enantiopure conductors (DM-EDT-TTF)_2_ClO_4_ (DM-EDT-TTF = dimethyl-ethylenedithio-tetrathiafulvalene) [6], motivated intensive endeavors toward the synthesis of a new series of chiral TTFs provided with various chiral groups and functionalities [7]. Concerning the latter, special attention has already been paid to the introduction of amide groups onto TTF donors to provide, for example, TTF monoamides [8,9,10,11], bis(TTF-amides) [12,13], or TTF-*ortho*-diamides [14,15,16], in order to take advantage of the hydrogen bonding donor–acceptor properties of the amides, which is beneficial in the crystal engineering of TTFs and their radical cation salts [17]. It is, thus, not surprising that a certain number of chiral TTF-amides have been synthesized since 2005, such as the following: the Me-EDT-TTF-diamides and DM-EDT-TTF-diamides, with one or two stereogenic centers on the ethylenedithio (EDT) bridge [18]; the *N*-(1′-phenylethyl)(BEDT-TTF)acetamide, with stereogenic centers both on the EDT bridge and on the *N*-substituent [19,20]; the EDT-TTF-mono- [21] and bis-hydroxyamides [22], which further provided EDT-TTF-oxazolines [23,24]; the binaphthyl-TTF-diamides with axial chirality [25]; the TTF-tetrakis(methyl-benzamides), which provides a conducting helical fiber [26] (Figure 1); or the benzene-1,3,5-tricarboxamides tris(TTF) precursors, which exhibit self-assembling into helical aggregates [27,28,29].

In the continuation of our research on chiral TTF precursors and materials, we report herein the synthesis, chiroptical, electrochemical, and structural characterization of the bis(TTF) derivatives **1** (Figure 1) bearing the 1,2-cyclohexane-diamide scaffold with two vicinal stereogenic centers. DFT calculations have been performed as well. Our main motivation concerning the chiral pincer-like redox active compound **1** was related to the possibility of accessing the intra- and intermolecular mixed-valence states in solution and in the solid state.

## 2. Results and Discussion

### 2.1. Synthesis and Characterization of Racemic and Enantiopure Donor 1

**Scheme 1:**
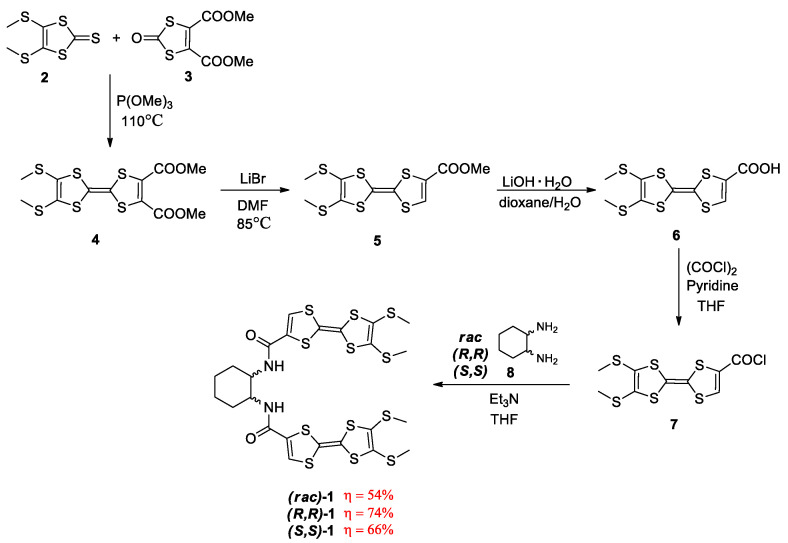
Synthesis of compounds **1**.

The racemic (*R*,*R*) and (*S*,*S*) forms of **1** were obtained following the multi-step synthetic strategy outlined in Scheme 1. Dithiole-thione **2** was engaged in a phosphite-mediated heterocoupling reaction with diester dithiolone **3** in order to obtain TTF-diester **4**. The latter was subjected to mono-decarboxylation to give **5**, then saponification of the remaining ester group to provide TTF-acid **6**, followed by a nucleophilic substitution to access acyl chloride **7**. Without any purification, the highly reactive acyl chloride **7** was engaged in a substitution reaction with 1,2-cyclohexanediamide **8**, as a racemic mixture or pure enantiomers (*R*,*R*) and (*S*,*S*), in THF, in the presence of Et_3_N (Scheme 1). The target diamides **1** were obtained in good yields (54 to 74%) after conducting chromatographic separation with an eluent mixture of dichloromethane/acetone = 4/0.1, and the formation of the desired compounds was confirmed by NMR spectroscopy (Figures S1–S5) and MS spectrometry. The 2D-COSY-NMR shows the signal for the CH protons of the cyclohexenyl unit at 3.84 ppm, coupled with the protons of the NH units from 7.04 ppm.

**Figure 2:**
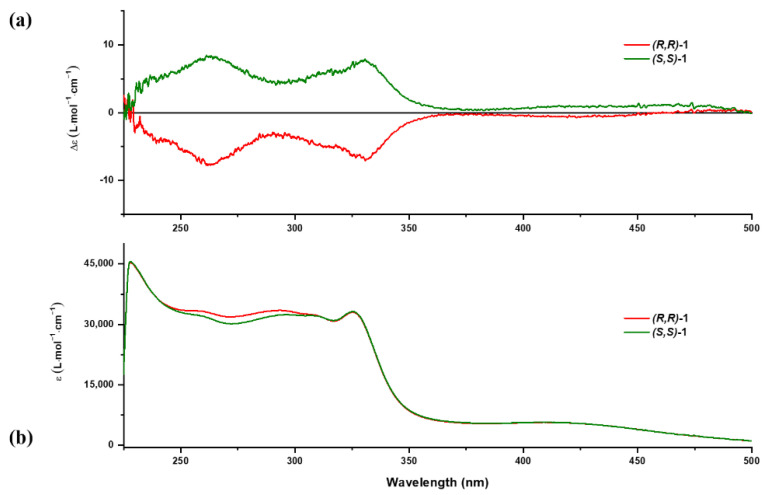
CD (**a**) and UV-Vis (**b**) spectra of compound **1** pair of enantiomers. Green line: (*S*,*S*) enantiomer, red line: (*R*,*R*) enantiomer.

The mirror-image circular dichroism (CD) spectra of (*R*,*R*)-**1** and (*S*,*S*)-**1** confirm their enantiomeric relationship, while the bands’ intensities suggest a relatively low chiroptical activity of these compounds (Figure 2a). The UV-Vis spectra show a broad, low-intensity band in the range of 360–490 nm, with a maximum absorption wavelength at 420 nm, followed by a 325 nm absorption maximum band and an absorption plateau in the range between 250–310 nm (Figure 2b).

**Figure 3:**
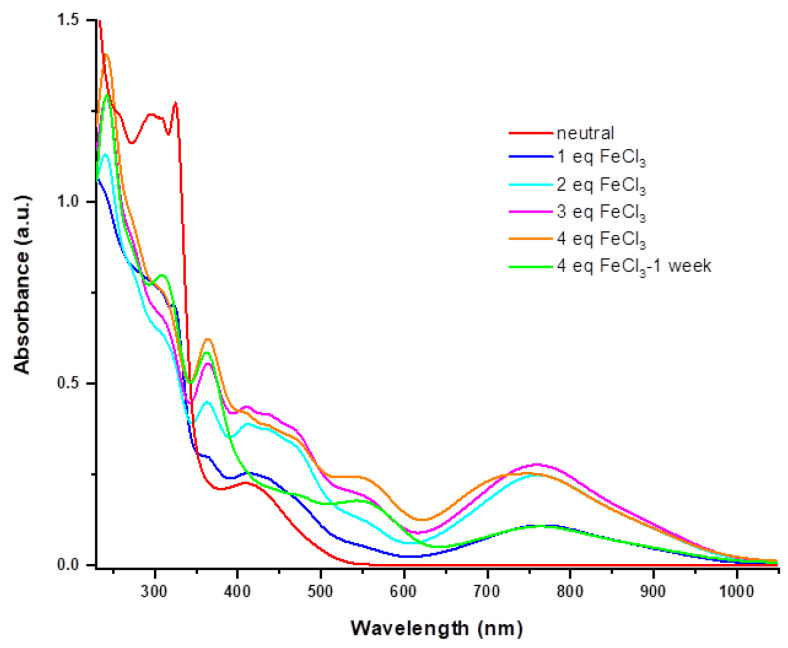
UV-Vis absorption spectra of (*rac*)-**1** during chemical oxidation by successive addition of FeCl_3_ in DCM at room temperature.

In order to investigate the absorption behavior of the oxidized (*rac*)*-***1**, its oxidation was performed chemically, using FeCl_3_ as an oxidant in a DCM solution at room temperature. The oxidation process, monitored through UV-Vis measurements (Figure 3), shows the emergence of two bands at 552 and 762 nm, typical of TTF^+**•**^, along with the decrease in intensity of the 325 nm band. After the addition of four equivalents of FeCl_3_, the oxidation process was complete. The oxidized species appeared quite stable, as suggested by the absorption spectra of the oxidized solution measured after one week (green line).

**Figure 4:**
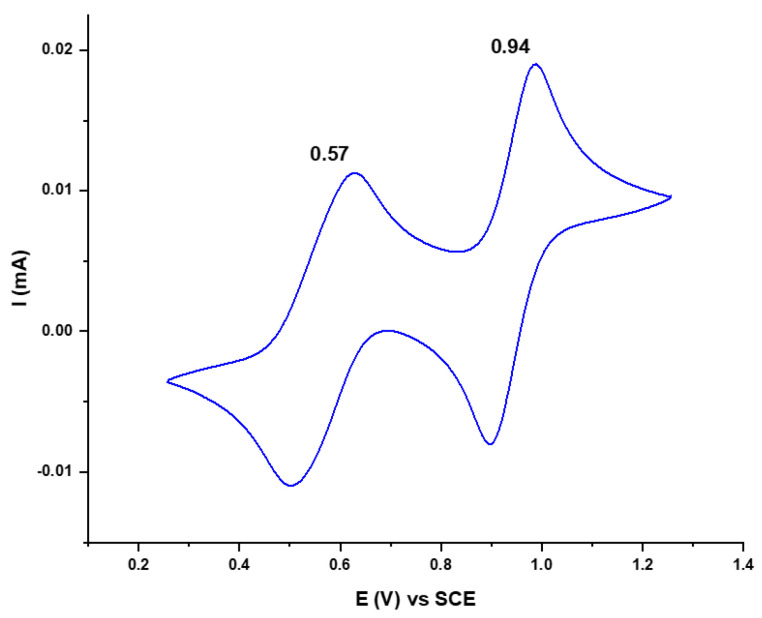
Cyclic voltammogram of (*rac*)-**1** in 0.1 M Bu_4_NPF_6_/CH_2_Cl_2_, Pt electrode, scan rate 100 mV s^−1^. The E_1/2_ values are shown for both reversible peaks.

The electronic properties of compound (*rac*)-**1** were investigated by cyclic voltammetry performed in solution at room temperature. The cyclic voltammogram shows the typical electrochemical behavior of TTF, with two reversible peaks, assigned to the formation of the radical cation and the dication, respectively, at ΔE_1/2_ = 0.57 and 0.94 V vs. SCE (Figure 4). In addition, the electrochemical experiment was carried out by varying the scan rate in order to investigate the possible multi-step oxidation of the two TTF moieties of the molecule, yet no peak splitting was observed. Indeed, in the solid-state structure (vide infra), the two TTF units of (*rac*)-**1** seem to be favorably disposed towards a through-space intramolecular interaction, which could be visible in the cyclic voltammogram via stepwise oxidation with the formation of mixed-valence [(TTF)_2_]^+•^ dimers. However, in solution, the two amido-TTF arms were flexible, thus providing a multitude of possible conformations (see also the DFT calculations Section) in which the TTF units do not interact with each other. 

**Figure 5:**
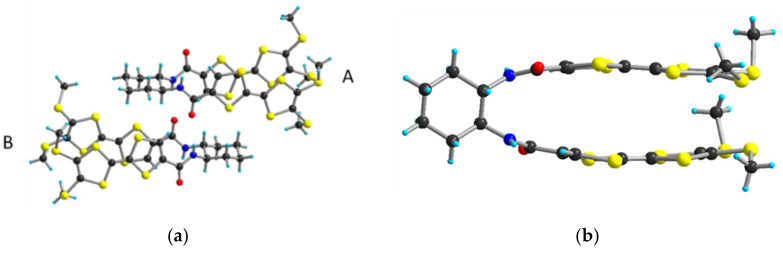
Asymmetric unit structure of (*rac*)-**1** (**a**) and top view of molecule **A** (**b**).

The racemic form of compound **1** gave narrow, plate-like, orange crystals suitable for X-ray measurements by slow evaporation of the solvent mixture (*v*/*v*, DCM/EE = 3/2). No structure could be determined for the enantiopure compounds, despite the formation of interesting hollow, tube-shaped crystals (Figure S6). Further, (*rac)-***1** crystallized in the centrosymmetric triclinic *P*–1 space group, with two independent molecules (**A** and **B**) in the asymmetric unit, arranged in a head-to-tail fashion. The TTF moieties of each molecule were disposed of in a face-to-face manner, forming pincer-like frameworks, while the oxygen atoms of the amide units were disposed in opposite directions and orthogonal to the cyclohexane plane (Figure 5).

**Figure 6:**
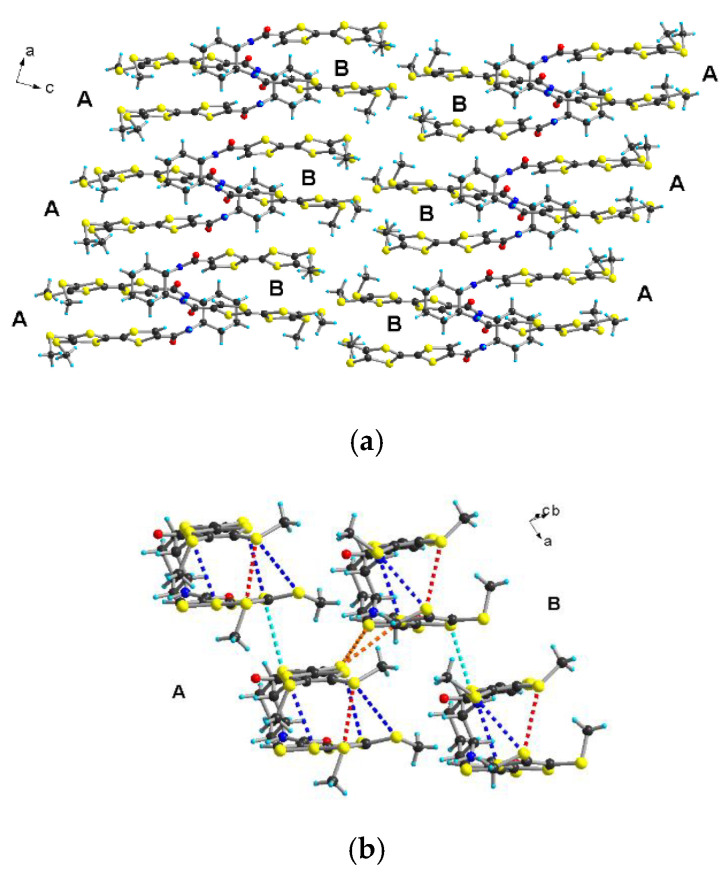
Packing of (*rac*)-**1** (**a**) in the *ac* plane and the intra- and intermolecular S···S short contacts (**b**).

In the crystal, the asymmetric unit (**AB**) forms columns along the *a*-axis, which alternate along the *c*-axis (Figure 6). Within the stack, molecules show a short S**···**S interaction of 4.01 Å for molecules **A,** and 3.72 Å for molecules **B** (turquoise dotted line), and of 3.86–4.06 Å between the **A** and **B** molecules (orange dotted lines). The pincer-like framework of the TTF units of each molecule is due to the S**···**S interactions of 3.81–3.98 Å (blue dotted lines) and of 3.38 Å for molecules **A** and 3.51 Å for molecules **B** (red dotted lines).

**Figure 7:**
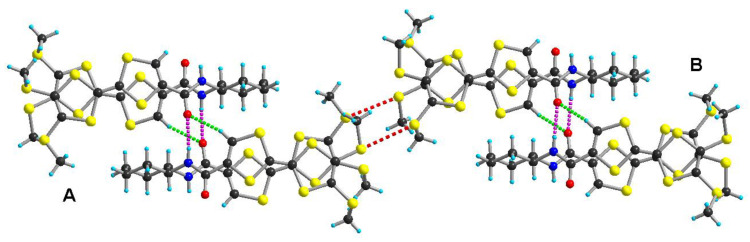
H bonding interaction between **A**- and **B**-type molecules.

Both the **A**- and **B**-type molecules interact laterally with each other by a hydrogen bonding N–H∙∙∙O type of 2.24–2.25 Å (magenta dotted lines) and C–H∙∙∙O type of 2.28–2.48 Å (green dotted lines). Moreover, the dimers of the AA and BB molecules further interact in a tail-to-tail fashion through the S**···**S short contacts of 3.46–3.58 Å (red dotted lines) (Figure 7).

### 2.2. Electrocrystallization of Donor 1

**Figure 8:**
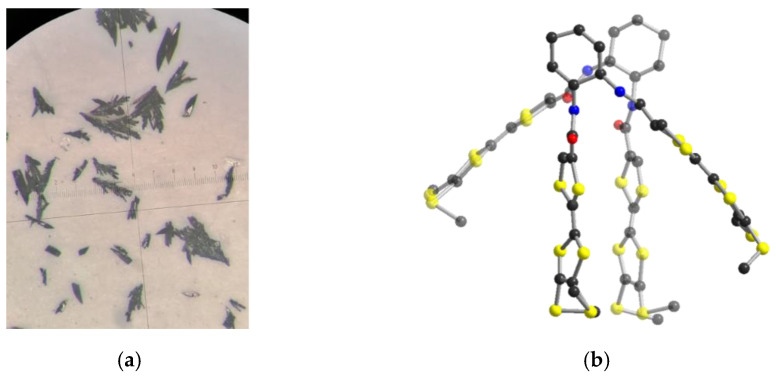
View of crystals (**a**) and the partial structure of (*rac*)-**1** in the radical cation salt with AsF_6_^−^ (**b**). The anions were omitted due to insufficient crystallographic data.

The three forms of **1** (*racemic*, *R*,*R* and *S*,*S*) were engaged in the electrocrystallization experiments, carried out with several anions, such as PF_6_^−^, AsF_6_^−^, ClO_4_^−^, ReO_4_^−^, and I_3_^−^. The enantiopure compounds did not form crystals by electrocrystallization, whereas the racemic form provided several radical cation salts under the same experimental conditions. Although well-defined crystals were obtained, indicating the formation of radical cation salts, their structure could not be determined as a consequence of the low quality of the crystals (Figure 7, left). Nevertheless, in the case of the AsF_6_^−^ material, a partial structure could be obtained, but the ratio between the donor and the anion in the asymmetric unit cell could not be established. The radical cation salt, containing the (*rac*)-**1** donor and the AsF_6_^−^ anion, crystallized in the centrosymmetric triclinic *P*–1 space group, with two independent donor molecules in the asymmetric unit cell and at least three anion molecules. Even though useful information, such as the donor–anion interactions, oxidation degree of the TTF units, or the inter-stack short contacts, could not be provided by this partial structure, an overview of the packing pattern of the donor molecules can be approximated. Therefore, as shown in Figure 8b, the TTF moieties were organized in a face-to-face manner, similar to the neutral form. In this case, the two TTFs belong to different donor molecules, and the intermolecular interactions take place in a head-to-head fashion. The segregation of the cyclohexane rings and the TTF units suggests interactions through hydrogen bonding between the amide groups of the arms of different molecules and between the amide groups and the TTF units. Still, this assumption cannot be confirmed by the present partial structure.

**Figure 9:**
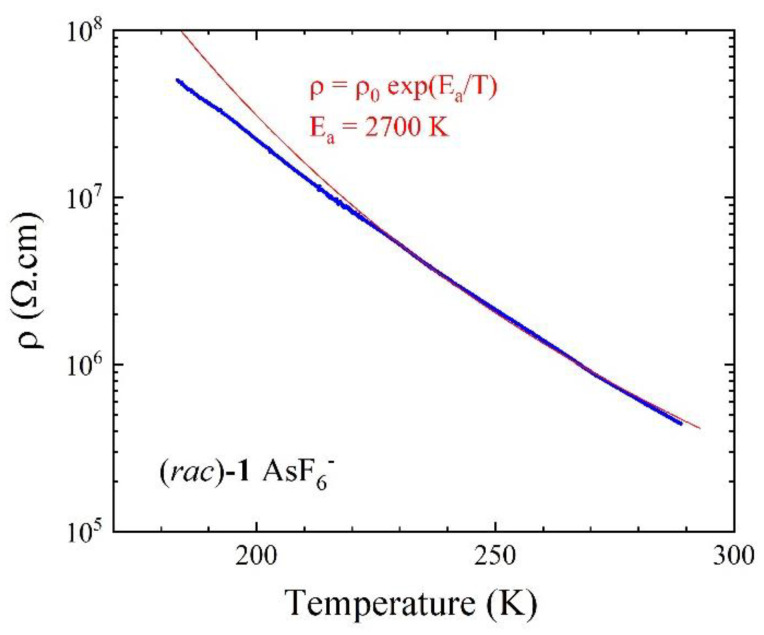
Temperature dependence of the electrical resistivity (blue line) for a single crystal of the radical cation salt of (rac)-**1** with AsF_6_^−^. The red line is the fit to the data with a law of the type ρ = ρ_0_exp(E_a_/T).

Resistivity measurements were performed on single crystals of the radical cation salt of (*rac*)-**1** and AsF_6_^−^, and the results are presented in Figure 9. The temperature dependence of the electrical resistivity indicates a semiconducting behavior of this radical cation salt, with a room temperature conductivity of 1.5–2∙10^−6^ S cm^−1^ and activation energy of 2700 K, as estimated to be in the range of 200–300 K.

### 2.3. DFT and TD-DFT Calculations

**Figure 10:**
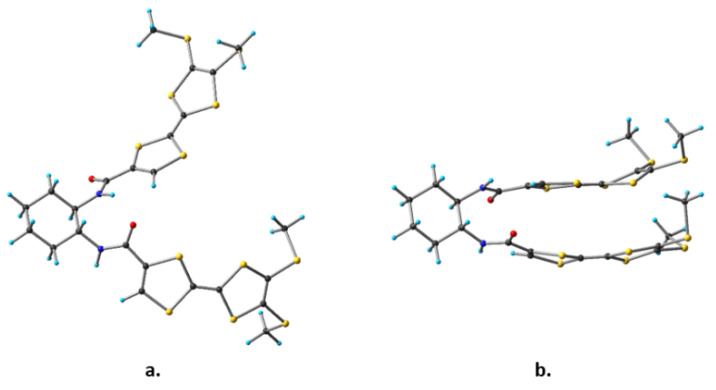
Equilibrium geometries of the neutral compound (*R*,*R*)-**1**, optimized at the following DFT levels: (**a**) B3LYP/Def2-SVPD, (**b**) B3LYP-D3/Def2-SVPD.

The structural features and optical properties of compound (*R*,*R*)-**1** were further investigated by using hybrid-DFT methods. The molecular geometry, optimized at the B3LYP/Def2-SVPD level of theory (Figure 10a), reveals few differences compared to the solid-state structure; however, the most striking one consists of a significantly larger intramolecular distance between the two pendant arms (i.e., TTF units) in the case of the former (see also Figure 5 for comparison). In another approach, by accounting for the long-range dispersion corrections in the DFT analyses (i.e., Grimme’s D3 empirical corrections), the equilibrium geometry of **1** becomes considerably more compact (Figure 10b), with the two TTF moieties exhibiting the same face-to-face stacking, as seen in the X-ray structure.

Even though the structure, optimized at the B3LYP-D3/Def2-SVPD level (Figure 10b), reproduces the solid-state one, TD-DFT performed on this geometry reveals only a poor agreement with the experimental chiro-optical properties. On the other hand, the structure obtained in the absence of the D3 corrections (Figure 10a) provides the CD and UV-Vis data in better agreement with the experimental measurements (see Figure S7 in the SI for a comparison). Therefore, we can conclude that the long-range dispersions are overestimated in this particular case, considering that, in solution, the TTF arms are most likely mobile in contrast to the rigid solid-state structure. The following will discuss only the case that best fits the chiro-optical experimental data.

**Figure 11:**
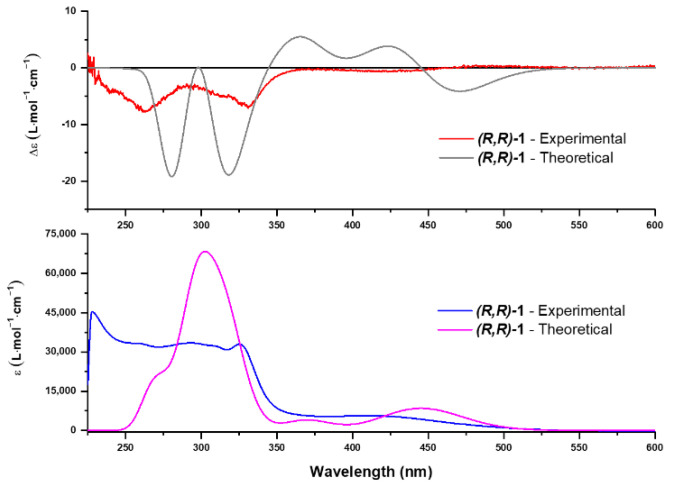
Experimental and theoretical (TD-B3LYP/Def2-SVPD) CD and UV-Vis spectra for neutral compound (*R*,*R*)-**1**.

**Figure 12:**
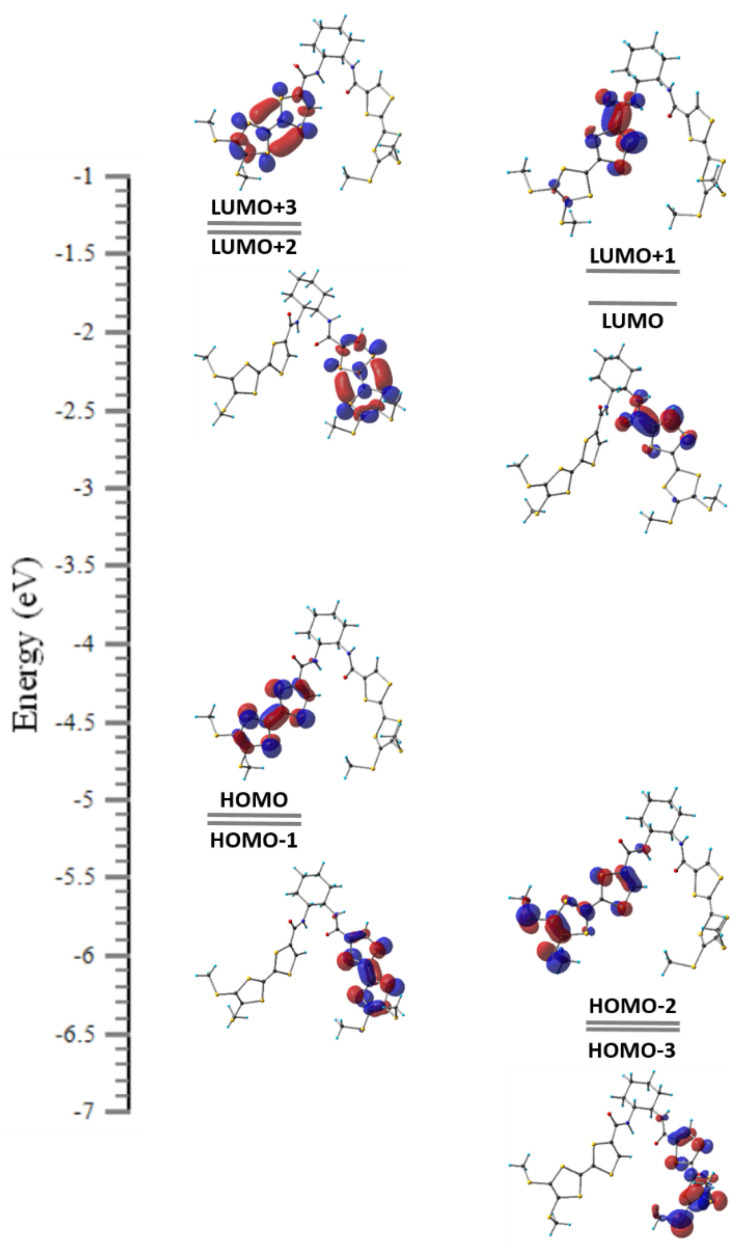
Most relevant frontier MOs involved in the monoelectronic excitations computed for compound (*R*,*R*)-**1** at the B3LYP/Def2-SVPD level of theory. Brief description of the MOs: HOMO-3 to HOMO: π orbital on the two TTF units; LUMO and LUMO+1: π* orbitals on the two different arms of compound (*R*,*R*)-**1**; LUMO+2 and LUMO+3: σ* orbital on the TTF units.

**Figure 13:**
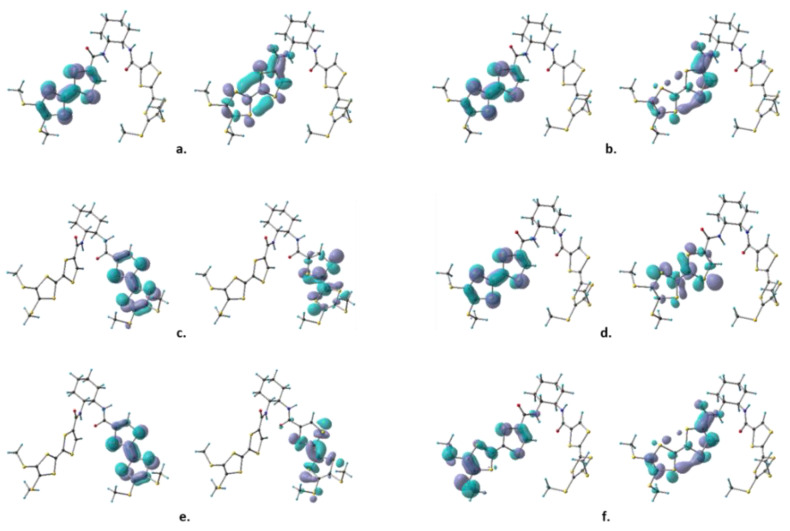
NTOs computed for the neutral (*R*,*R*)-**1** enantiomer, describing the following monoelectronic excitations: (**a**) ***S0 → S2***, (**b**) ***S0 → S4***, (**c**) ***S0 → S13***, (**d**) ***S0 → S14***, (**e**) ***S0 → S16*** and (**f**) ***S0 → S25***.

The comparisons between the experimental and calculated (TD-B3LYP/Def2-SVPD) absorption and CD spectra of (*R*,*R*)-**1** are illustrated in Figure 11. The theoretical CD spectrum is in agreement with the measured one, especially in the high-energy range. As for the 500–350 nm region, a possible explanation for the lack of a clear experimental CD band could be rooted in the complex conformational equilibria occurring in the solution. The selection of the most relevant excited states, computed for the investigated (*R*,*R*)-**1** enantiomer, is presented in Table S1. In short, the monoelectronic excitation ***S0*** → ***S1*** explains the negative CD wave of the TD-DFT spectrum, centered at ca. 475 nm, but also reveals a signal of moderate-to-low intensity in the absorption spectrum. In terms of the molecular orbitals (MOs), it mainly involves the HOMO-1 → LUMO transition, i.e., a π–π* excitation occurring on one of the two TTF arms (see Figure 12 for the shape and localization of the frontier MOs). The second excited state affords the positive CD band of the theoretical spectrum, centered at ca. 425 nm, while in UV-Vis, this second transition displays—similar to the first excitation—a moderate-to-low intensity signal. From a MO perspective, ***S0*** → ***S2*** is more complex than the previous one because it is described by two sets of canonical orbitals that involve the following transitions: HOMO → LUMO+1 and HOMO → LUMO+3 (see also Table S1). To gain further insight into this hole-particle excitation, natural transition orbital (NTO) explorations have been additionally performed. Based on this technique, ***S0*** → ***S2*** is characterized as the transition from a π orbital into a mixed σ/π one, which occurs on one of the TTF arms (Figure 13a). Concerning the excitations ***S0*** → ***S3*** and ***S0*** → ***S4***, they exhibit low-intensity CD signals. However, the latter reveals a moderate-to-low optical activity in the absorption spectrum and involves the following sets of MOs: HOMO → LUMO+3, HOMO → LUMO+1, HOMO → LUMO. This monoelectronic excitation is also better understood in the light of NTO analysis, according to which it represents the π-to-σ/π transition at the level of one of the TTF moieties (Figure 13b). The excitations ***S0*** → ***S7*** and ***S0*** → ***S8*** are also relevant (Table S1), given that along with ***S0*** → ***S1***, ***S0*** → ***S2*** and ***S0*** → ***S4***, they explain the broad adsorption band of the relatively low intensity observed at lower energies in the measured spectrum (between ca. 500 and 350 nm).

Other important excitations are ***S0*** → ***S13***, ***S0*** → ***S14***, ***S0*** → ***S16*** and ***S0*** → ***S25*** (Table S1). Among these transitions, ***S0*** → ***S13*** and ***S0*** → ***S14*** explain the intense adsorption experimental maximum band at 325 nm, while ***S0*** → ***S13***, ***S0*** → ***S16*** and ***S0*** → ***S25*** account for the negative CD waves centered at ca. 325 ppm and 275 nm, respectively. The NTO pairs describing these excitations are illustrated in Figure 13.

## 3. Materials and Methods

All commercially available reagents and solvents were used as received unless otherwise noted. Dry tetrahydrofuran was directly used from the purification machines. The chromatography purifications were performed on silica gel Sorbent Technologies Silica Gel (60 Å, 65 × 250 mesh), and thin-layer chromatography (TLC) was carried out using aluminum sheets precoated with silica gel 60 (EMD 40–60 mm, 230–400 mesh with 254 nm dye). The NMR spectra were acquired with the Bruker Avance DRX 300 and 500 spectrometers operating at 300 and 500 MHz for ^1^H and 75 and 125 MHz for ^13^C, respectively, at room temperature in CDCl_3_ solutions. The ^1^H and ^13^C NMR spectra were referenced to the residual protonated solvent (^1^H) or the solvent itself (^13^C). All chemical shifts are expressed in parts per million (ppm) downfield from external tetramethylsilane (TMS), using the solvent residual signal as an internal standard, and the coupling constant values (J) are reported in Hertz (Hz). The following abbreviations have been used: s, singlet; br s, broad singlet; d, doublet; m, multiplet. Mass spectrometry MALDI–TOF MS spectra were recorded on a Bruker Biflex-IIITM apparatus equipped with a 337-nm N_2_ laser. Elemental analyses were recorded using the Flash 2000 Fisher Scientific Thermo Electron analyser. The UV-Vis-NIR absorption spectra were recorded using a Perkin Elmer L950 spectrometer in CH_2_Cl_2_ solutions. The electrochemical experiments were performed with a SP150 Biologic Potentiostat in a standard three-electrode cell using platinum working disk electrodes of 2 mm diameters and 0.1 M TBAPF_6_ in dichloromethane as the supporting electrolyte, with a scan rate of 100 mV/s. Ferrocene has been used as an internal reference, and the values are reported versus the standard calomel electrode (SCE). Compounds **4**, **5** and **6** have been prepared by following the procedures described in [28,30].

(*rac*)-Cyclohexane-bis(TTF)-diamide (*rac*)-**1**: 250 mg (0.73 mmol) of carboxylic acid **6** was suspended in THF, and 0.19 mL (300 mg, 2.2 mmol) of oxalyl chloride was added dropwise in the presence of pyridine. The mixture was stirred at 45 °C for two hours under an inert atmosphere. The residue was evaporated to dryness (maintaining the inert atmosphere). The acyl chloride was solubilized in THF, and a solution of 0.03 mL (27 mg, 0.23 mmol) of (*rac*)-1,2-diaminocyclohexane and 0.03 mL (23 mg, 0.23 mmol) of Et_3_N in THF was added dropwise. The mixture was stirred overnight at room temperature. After the evaporation of THF, water was added and extracted with DCM. The organic phase was dried over MgSO_4_, and the residue was separated through column chromatography (silica gel, dichloromethane/acetone = 4/0.1). Yield: 54%; aspect: orange precipitate. ^1^H NMR (300 MHz, CDCl_3_) δ ppm: 7.04 (s, 2H), 6.82 (br s, 2H), 3.84 (m, 2H), 2.40 (m, 12H), 2.06 (d, *J* = 11.1 Hz, 2H), 1.83 (d, *J* = 6.8 Hz, 2H), 1.52–1.23 (m, 4H). ^13^C NMR (76 MHz, CDCl_3_): 160.39, 132.22, 128.07, 127.42, 126.17, 112.36, 109.48, 54.68, 31.99, 24.94, 19.43. MALDI-TOF MS: *m*/*z* = 759.0 [1]^+^. Elemental analysis calcd for C_24_H_26_N_2_O_2_S_12_: C, 37.97; H, 3.45; S, 50.67; N, 3.69. Found: C, 38.00; H, 3.35; S, 50.63; N, 3.58.

(*R*,*R*)-Cyclohexane-bis(TTF)-diamide (*R*,*R*)-**1**: The procedure is the same as above except that (*R*,*R*)-1,2-diaminocyclohexane has been used. Yield: 74%; aspect: orange precipitate. ^1^H NMR (300 MHz, CDCl_3_) δ ppm: 6.93 (s, 2H), 3.86 (s, 2H), 2.43 (s, 12H), 2.05 (d, *J* = 11.4 Hz, 2H), 1.83 (d, *J* = 7.2 Hz, 2H), 1.51–1.24 (overlapped signals, 4H). HRMS (TOF) calcd for C_24_H_26_N_2_O_2_S_12_ [M+]: 757.86373, found 757.86330 (error −0.57 ppm).

(*S*,*S*)-Cyclohexane-bis(TTF)-diamide (*S*,*S*)-**1**: The procedure is the same as above except that (*S*,*S*)-1,2-diaminocyclohexane has been used. Yield: 66%; aspect: orange precipitate. ^1^H NMR (300 MHz, CDCl_3_) δ ppm: 6.67 (s, 2H), 3.81 (s, 2H), 2.42 (s, 12H), 2.08 (d, *J* = 10.0 Hz, 2H), 1.83 (s, 2H), 1.43–1.25 (overlapped signals, 4H). HRMS (TOF) calcd for C_24_H_26_N_2_O_2_S_12_ [M+]: 757.86330, found 757.86390 (error 0.23 ppm).

Details about the data collection and solution refinement are given in Table 1. Single crystals of the compounds were mounted on glass fiber loops using a viscous hydrocarbon oil to coat the crystal and then were transferred directly to a cold nitrogen stream for data collection. X-ray single-crystal diffraction data were collected at 150 K on an Agilent SuperNova diffractometer equipped with an Atlas CCD detector and mirror-monochromated micro-focus Cu-K_α_ radiation (λ = 1.54184 Å). The structure was solved by direct methods and expanded and refined on F^2^ by the full-matrix least-squares techniques using SHELX 2018 programs (G.M. Sheldrick, 2016). All non-H atoms were refined anisotropically, and the H atoms were included in the calculation without refinement. Multiscan empirical absorption was corrected using the CrysAlisPro program (CrysAlisPro, Agilent Technologies, V1.171.37.35g, 2014). The crystallographic data of the structure obtained at 150 K has been deposited with the Cambridge Crystallographic Data Centre, deposition number CCDC 2206330. These data can be obtained free from CCDC, 12 Union Road, Cambridge CB2 1EZ, UK (e-mail: deposit@ccdc.cam.ac.uk or http://www.ccdc.cam.ac.uk, accessed on 1 August 2022). A summary of the crystallographic data and the structure refinement for the single crystals of compounds **1**–**4** is given in Table 1.

The electrical resistivity experiments were performed at two points on 0.2 to 0.3 mm-long single crystals of the radical cation salt of (*rac*)-**1** with AsF_6_⁻. Gold wires were glued with silver paste on both ends of the crystals. The temperature dependence of the resistivity was measured by applying a constant voltage (20 V) and measuring the current with a Keithley 486 picoammeter. The resistivity value has also been checked at room temperature by applying a DC current (0.01 µA) and measuring the voltage with a Keithley 2400 source meter. The low temperature was provided by liquid nitrogen in a homemade cryostat.

### Computational Details

Software: all calculations were performed with the *Gaussian 09* package [30].

DFT calculations: the molecular geometries of the investigated species were fully optimized without any symmetry constraints in dichloromethane (DCM), employing the SMD variation [31] of the polarizable continuum solvation model (e.g., for DCM, ε = 8.93). In all DFT calculations, the B3LYP [32,33] hybrid functional was employed, along with Ahlrichs’ double-zeta quality basis set, Def2-SVPD [34,35], augmented by the polarization and diffuse functions on all atoms. Based on our previous experience with TTF derivatives [36], hybrid-DFT methods, coupled with augmented basis sets, are accurate enough to reproduce the experimental molecular geometries of such species. Moreover, Grimme’s D3 empirical corrections [37] were included in the DFT calculations (i.e., such calculations are referred to as B3LYP-D3/Def2-SVPD throughout the text) to test whether long-range dispersion affects the computed structural features of the investigated derivatives. In all computations, the optimization criteria were set to tight. The calculations of the vibrational frequencies were systematically performed to characterize the nature of the stationary points. According to this analysis, all optimized geometries are true minima on the potential energy surface (PES). The integration grid used in all calculations comprised 99 radial shells and 950 angular points for each shell (99,950), also known as the “ultrafine” grid in Gaussian 09.

TD-DFT calculations: time-dependent (TD) DFT investigations were performed as *single-point* calculations on the previously optimized structures in DCM (accounting for the same SMD variation in the polarizable continuum solvation model). According to the seminal paper of Pescitelli and Bruhn, [38] TD-DFT calculations employing hybrid functionals (i.e., such as B3LYP) in conjunction with double-zeta quality basis sets, which are augmented by both polarization and diffuse functions (i.e., such as the Def2-SVPD basis set), generally give reliable results in terms of the chiroptical properties. The calculated number of singlet monoelectronic excitations was 40 in all cases. The length gauge formulation was employed for the calculation of the rotatory strengths. Nevertheless, the velocity gauge formulation leads to roughly the same values as the rotatory strengths. Computed spectra were plotted with the *SpecDis* tool [38,39] and were enlarged by a Gaussian shape displaying a full width at half-maximum (FWHM) of 0.2 eV (for both CD and UV-Vis plots) to compare with the experimental data. 

NTO analysis: natural transition orbitals (NTOs) [40] are generally a very useful tool for describing electronic excitations. This type of analysis allows a localized picture of the complex transitions involving multiple sets of canonical molecular orbitals. In fact, the NTO description is much more intuitive than the MO-based one, given that complex excited states can usually be expressed as single pairs of NTO orbitals. In the present study, the NTOs were generated with the *Multiwfn* program [41].

## 4. Conclusions

The bis(tetrathiafulvalene)-1,2-cyclohexane-diamide donor **1** has been synthesized as enantiopure (*S*,*S*) and (*R*,*R*) forms together with the racemic mixture, starting from the corresponding cyclohexane-diamines and TTF-acid chloride precursors. In the solid-state structure, the donor adopts a pincer-like configuration, which is favorable to promoting through-space communication between the two TTF units and, thus, mixed-valence species. However, the cyclic voltammetry measurements do not seem to indicate the splitting of the oxidation waves. In agreement with these findings, the molecular orbital diagram shows degenerate frontier orbitals located on separate TTFs. The TD-DFT calculations explain very well the electronic excitfations observed in the experimental UV-Vis and CD spectra of **1**. Chemical oxidation of the donor allows the observation of the formation of stable radical cation species of **1**. The electrocrystallization experiments afforded a crystalline radical cation salt of (*rac*)-**1** with the AsF_6_^−^ anion, showing semiconducting behavior according to the single-crystal resistivity measurements. The results reported herein open the way towards the use of such chiral bis(TTF) donors to prepare original molecular conductors. Variations in the TTF unit’s substitution pattern can be envisaged to tune the crystallization propensity of the precursors and their charge transfer and radical cation salts.

## Figures and Tables

**Table 1:** Crystallographic data, details of data collection and structure refinement parameters for compound (*rac*)-**1**.

	(*rac*)-1
Formula	C_24_H_24_N_2_O_2_S_12_
M [g∙mol^−1^]	757.17
*T* [K]	150.01(10)
Crystal system	triclinic
Space group	*P*–1
*a* [Å]	9.0042(7)
*b* [Å]	16.8256(13)
*c* [Å]	21.7297(10)
*α* [°]	78.082(5)
*β* [°]	85.629(5)
*γ* [°]	80.197(6)
*V* [Å^3^]	3171.2(4)
Z	4
*ρ*_calcd_ [g cm^−3^]	1.586
*μ* [mm^−1^]	7.917
Goodness-of-fit on *F*^2^	1.183
Final *R_1_^a^*/w*R_2_^b^ [I > 2σ(I)]*	0.1109/0.3032
*R_1_^a^*/w*R_2_^b^* (all data)	0.1708/0.4094
CCDC number	2206330

^a^ R_1_ = ∑||F_o_|–|F_c_||/∑|F_o_|. ^b^ wR_2_ = [∑w(F_o_^2^ *−* F_c_^2^)^2^/∑w(F_o_^2^)^2^]^1/2^; w = 1/[*σ*²(F_0_^2^) + (aP)^2^ + bP] where P = [max(F_0_^2^, 0) + 2F_c_^2^]/3.

## Data Availability

Not applicable.

## Supplementary Materials

The following are available online at https://www.mdpi.com/article/10.3390/molecules27206926/s1, Figure S1: ^1^H-NMR spectrum of compound (*rac*)-**1**, Figure S2: ^13^C-APT-NMR spectrum of compound (*rac*)-**1**, Figure S3: 2D-COSY-NMR spectrum of compound (*rac*)-**1**, Figure S4: ^1^H-NMR spectrum of compound (*R*,*R*)-**1**, Figure S5: ^1^H-NMR spectrum of compound (*S*,*S*)-**1**, Figure S6: Example of hollow tubular crystals of enantiopure **1** and the asymmetric unit structure of (*rac*)-**1**, Figure S7: Experimental and theoretical (TD-B3LYP/Def2-SVPD and TD-B3LYP-D3/Def2-SVPD) CD and UV-Vis spectra determined for the neutral form of enantiomer (*R*,*R*)-**1**, Table S1: Selection of the most relevant excited singlet states computed for the neutral compound (*R*,*R*)-**1**. The calculated oscillator and rotatory strengths, along with the molecular orbital descriptions of these excitations, are displayed.

## Sample Availability

Samples of the compounds are not available from the authors.
