# Supplementary material for: Chiral Bis(tetrathiafulvalene)-1,2-cyclohexane-diamides
Source: Molecules. 2022 Oct 15;27(20):6926. doi: 10.3390/molecules27206926 (PMC9611696; doi:10.3390/molecules27206926)
Supplement: Supplementary file 1 [file molecules-27-06926-s001.zip › molecules-1951240-supplementary.pdf]

Supporting information for

Chiral Bis(tetrathiafulvalene)-1,2-Cyclohexane-diamides

Alexandra Bogdan [1,2], Ionuț-Tudor Moraru [2], Pascale Auban-Senzier [3], Ion Grosu [2], Flavia Pop [1,*] and Narcis Avarvari [1,*]

[1] Univ Angers, CNRS, MOLTECH-Anjou, SFR MATRIX, F-49000 Angers, France;
[2] Faculty of Chemistry and Chemical Engineering, Department of Chemistry, Babes-Bolyai University, Cluj-Napoca, 11 Arany Janos Str., 400028 Cluj-Napoca, Romania
[3] Laboratoire de Physique des Solides, Université Paris-Saclay CNRS UMR 8502, Bât. 510, F-91405 Orsay, France
* Correspondence: flavia.pop@univ-angers.fr (F.P.); narcis.avarvari@univ-angers.fr (N.A.)

NMR characterization of 1

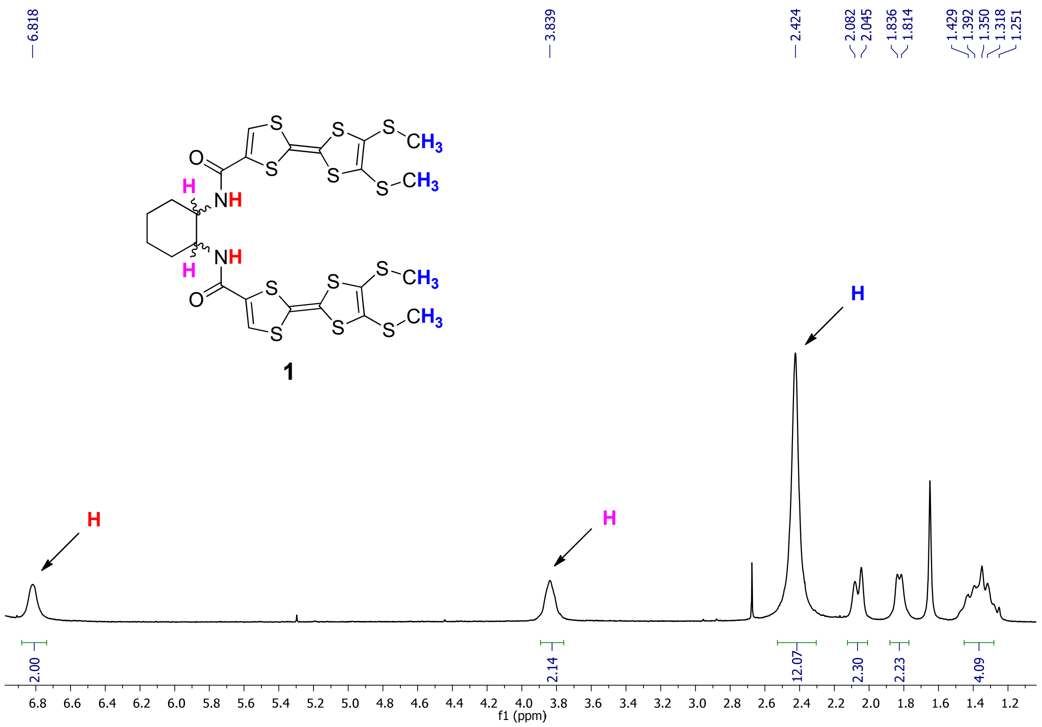

Figure S1. Fragment of the ^{1}H-NMR spectrum (300 MHz, $CDCl_3$) for compound **1**.

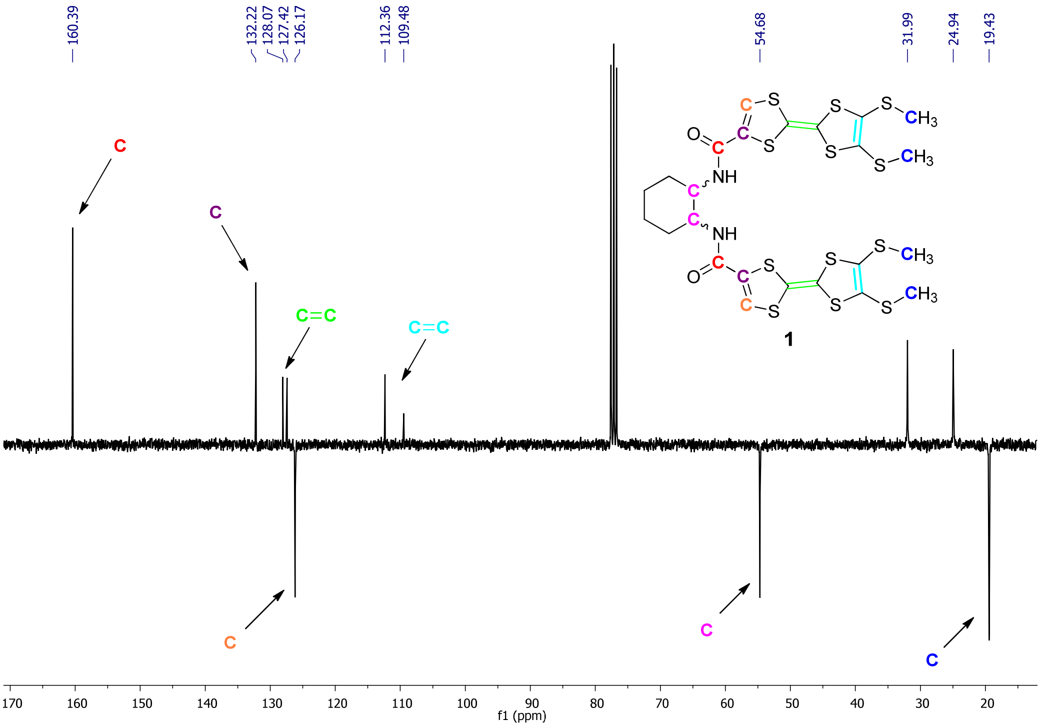

Figure S2. ^{13}C-APT-NMR spectrum (75 MHz, $CDCl_3$) of compound **1**.

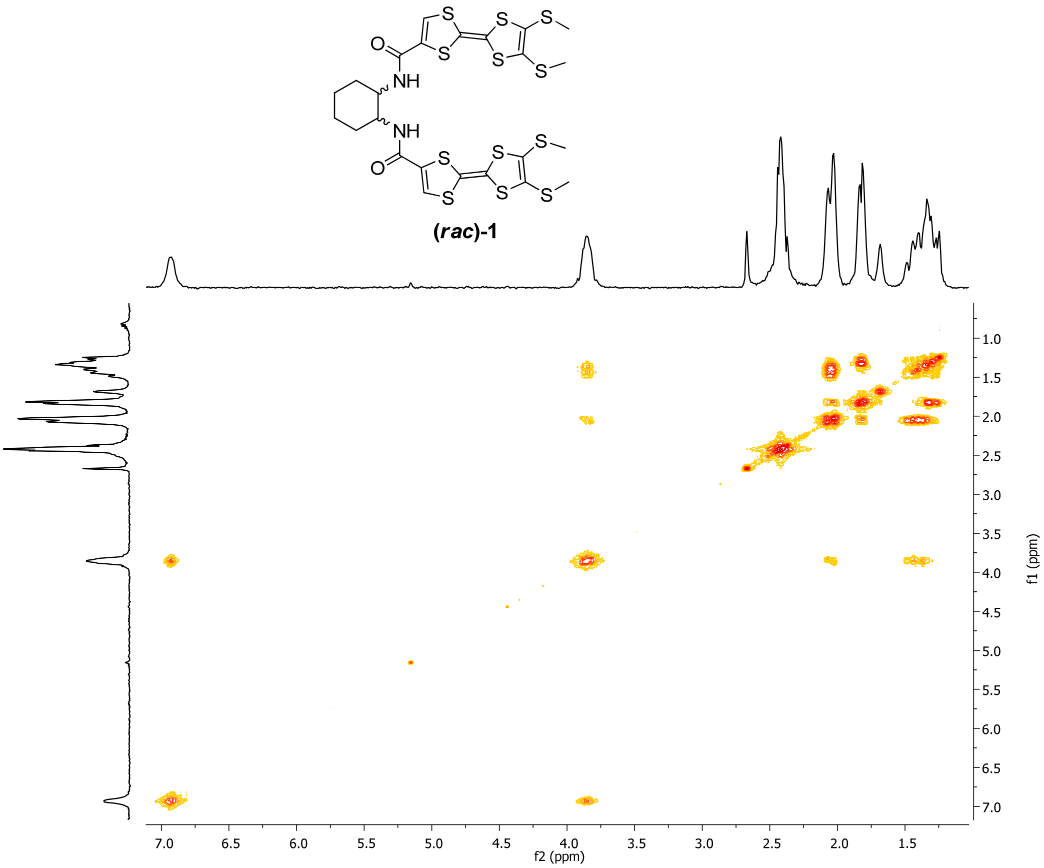

Figure S3. 2D-COSY-NMR spectrum (300 MHz, $CDCl_3$) of compound **1**.

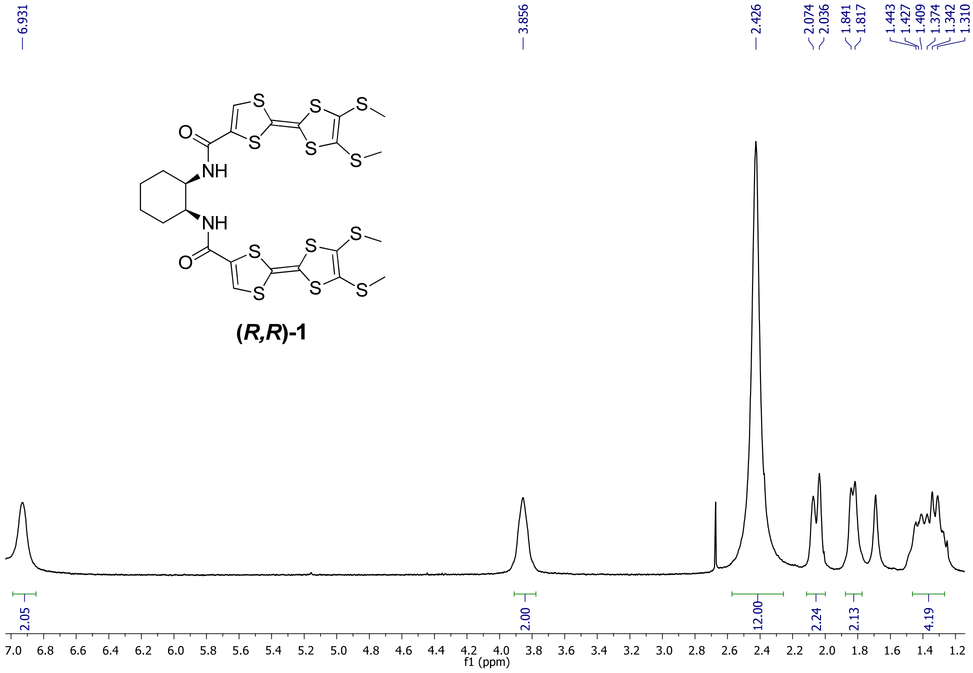

Figure S4. Fragment of ^{1}H-NMR spectrum (300 MHz, $CDCl_3$) for compound (*R*,*R*)-**1**.

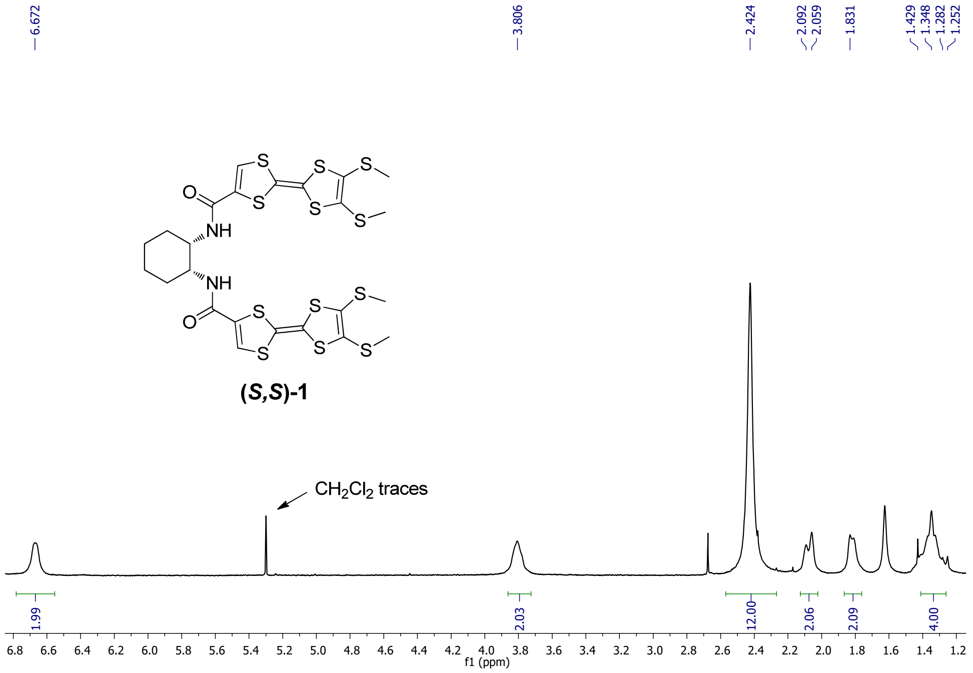

Figure S5. Fragment of ^{1}H-NMR spectrum (300 MHz, $CDCl_3$) for compound (*S*,*S*)-**1**.

Single crystal X-ray structures

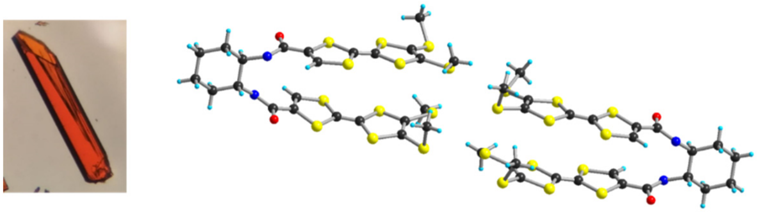

Figure S6. Example of hollow tubular crystals of enantiopure **1** (left) and the asymmetric unit structure of (*rac*)-**1** (right).

DFT and TD-DFT investigations

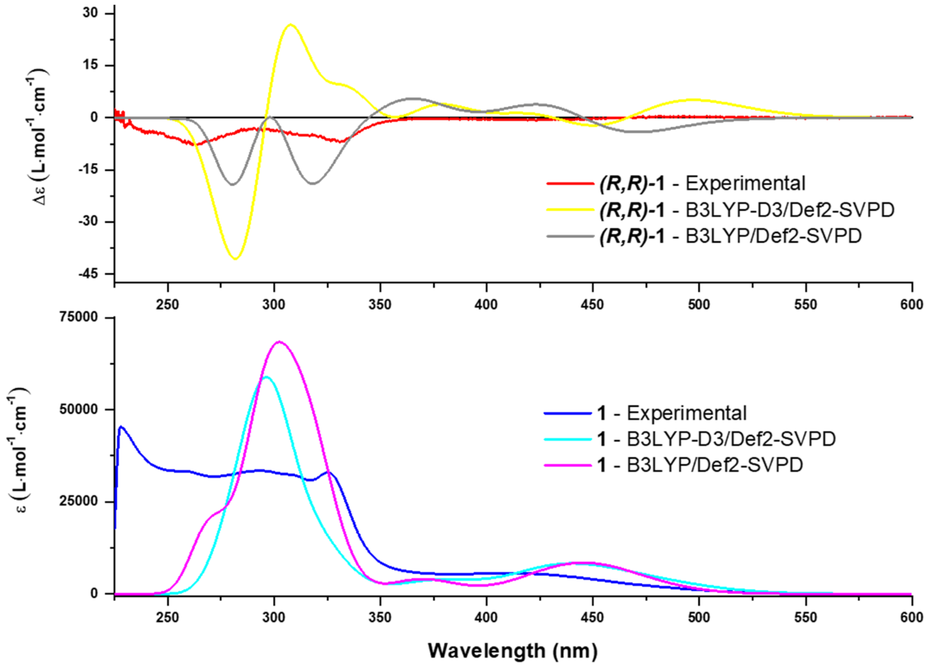

Figure S7. Experimental and theoretical (TD-B3LYP/Def2-SVPD and TD-B3LYP-D3/Def2-SVPD) CD and UV-Vis spectra determined for the neutral form of enantiomer (*R,R*)-**1.**

Table S1. Selection of the most relevant excited singlet states computed for the neutral compound **1**. The calculated oscillator and rotatory strengths along with the molecular orbital descriptions of these excitations are displayed.

No.	Wavelength (nm)	Oscillator Strength	Rotatory Strength	Orbital Contributions
S0→S1	456.9	0.0583	-25.1327	HOMO-1→ LUMO (**92%**), HOMO-1→ LUMO+2 (**7%**)
S0→S2	439.9	0.0006	18.2484	HOMO→ LUMO+1 (**52%**), HOMO→ LUMO+3 (**43%**)
S0→S3	432.2	0.0051	1.5936	HOMO-1→ LUMO+2 (**89%**), HOMO-1→ LUMO (**7%**), HOMO-1→ LUMO+1 (**2%**)
S0→S4	424.9	0.0230	4.2372	HOMO→ LUMO+3 (**52%**), HOMO→ LUMO+1 (**40%**), HOMO→ LUMO (**7%**)
S0→S7	371.1	0.0219	-18.2487	HOMO→ LUMO+5 (**97%**)
S0→S8	368.5	0.0270	30.6412	HOMO-1→ LUMO+4 (**98%**)
S0→ S13	317.8	0.1562	-85.2482	HOMO-1→ LUMO+6 (**36%**), HOMO→ LUMO+7 (**24%**), HOMO-3→ LUMO+2 (**8%**), HOMO-1→ LUMO+8 (**7%**), HOMO-1→ LUMO+9 (**5%**), HOMO-2→ LUMO+3 (**4%**), HOMO→ LUMO+9 (**4%**), HOMO→ LUMO+8 (**2%**), HOMO→ LUMO+10 (**2%**)
S0→ S14	316.9	0.3022	53.6623	HOMO→LUMO+7 (**34%**), HOMO-1→LUMO+6 (**21%**), HOMO-2→LUMO+3 (**8%**), HOMO→LUMO+9 (**8%**), HOMO-3→LUMO+2 (**5%**), HOMO-1→LUMO+8 (**6%**), HOMO-1→LUMO+9 (**3%**), HOMO→LUMO+8 (**3%**), HOMO→LUMO+10 (**2%**)
S0→ S16	304.2	0.0722	-127.4719	HOMO-1→ LUMO+8 (**38%**), HOMO-1→ LUMO+11 (**18%**), HOMO-1→ LUMO+6 (**8%**), HOMO-1→ LUMO+9 (**8%**), HOMO→ LUMO+9 (**7%**), HOMO→ LUMO+12 (**6%**), HOMO→ LUMO+8 (**5%**), HOMO→ LUMO+7 (**3%**), HOMO-3→ LUMO (**2%**)
S0→ S25	282.8	0.0125	-80.8797	HOMO-2→ LUMO+3 (**37%**), HOMO-2→ LUMO+1 (**30%**), HOMO→ LUMO+12 (**11%**), HOMO→ LUMO+9 (**6%**), HOMO→ LUMO+8 (**3%**), HOMO-2→ LUMO+5 (**2%**), HOMO→ LUMO+15 (**2%**), HOMO→ LUMO+16 (**2%**)
