# Supplementary material for: Chiral Bis(tetrathiafulvalene)-1,2-cyclohexane-diamides
Source: Molecules. 2022 Oct 15;27(20):6926. doi: 10.3390/molecules27206926 (PMC9611696; doi:10.3390/molecules27206926)

checkCIF/PLATON report

Structure factors have been supplied for datablock(s) abf43

THIS REPORT IS FOR GUIDANCE ONLY. IF USED AS PART OF A REVIEW PROCEDURE FOR PUBLICATION, IT SHOULD NOT REPLACE THE EXPERTISE OF AN EXPERIENCED CRYSTALLOGRAPHIC REFEREE.

No syntax errors found. CIF dictionary Interpreting this report

Datablock: abf43

```
Bond precision:   C-C = 0.0149 A          Wavelength=1.54184

Cell:           a=9.0042(7)        b=16.8256(13)     c=21.7297(10)
                alpha=78.082(5)    beta=85.629(5)    gamma=80.197(6)
Temperature:    150 K

                 Calculated                   Reported
Volume           3171.2(4)                    3171.2(4)
Space group      P -1                         P -1
Hall group       -P 1                         -P 1
Moiety formula   C24 H26 N2 O2 S12            C24 H26 N2 O2 S12
Sum formula      C24 H26 N2 O2 S12            C24 H26 N2 O2 S12
Mr               759.19                       759.19
Dx,g cm-3        1.590                        1.590
Z                4                            4
Mu (mm-1)        7.917                        7.917
F000             1568.0                       1568.0
F000'            1586.48
h,k,lmax         11,21,27                     11,19,26
Nref             13133                        10287
Tmin,Tmax        0.468,0.700                  0.802,1.000
Tmin'            0.167

Correction method= # Reported T Limits: Tmin=0.802 Tmax=1.000
AbsCorr = MULTI-SCAN

Data completeness= 0.783           Theta(max)= 75.270

R(reflections)= 0.1098( 5380)                    wR2(reflections)=
                                                 0.4096( 10287)
S = 1.183                  Npar= 705
```

The following ALERTS were generated. Each ALERT has the format
test-name_ALERT_alert-type_alert-level.
Click on the hyperlinks for more details of the test.

Alert level A

```
PLAT029_ALERT_3_A _diffrn_measured_fraction_theta_full value Low .      0.846 Why?
```

Author Response: The crystal was very small and despite long exposure time, the intensities were low or not observable at theta 66.67 degrees.

Alert level B

```
PLAT084_ALERT_3_B High wR2 Value (i.e. > 0.25) ...................       0.41 Report
```

Author Response: The model is correct but the overall quality of the data were poor due to the crystal quality. The crystal was very weakly diffracting at high angle and we can not find a better crystal.

```
PLAT196_ALERT_1_B No TEMP record and _measurement_temperature .NE.        293 Degree
PLAT230_ALERT_2_B Hirshfeld Test Diff for    S6A      --C12A     .        7.2 s.u.
```

Author Response: This can show possible disorder but the disordered model does not help in the modeling of this crystal.

```
PLAT340_ALERT_3_B Low Bond Precision on  C-C Bonds ..............    0.01493 Ang.
```

Author Response: The model is correct but the overall quality of the data were poor due to the crystal quality.

```
PLAT911_ALERT_3_B Missing FCF Refl Between Thmin & STh/L=    0.600       1752 Report
```

Author Response: This is most probably related to the low completness.

Alert level C

```
PLAT082_ALERT_2_C High R1 Value .................................       0.11 Report
PLAT112_ALERT_2_C ADDSYM Detects New (Pseudo) Symm. Elem         C         85 %Fit
PLAT213_ALERT_2_C Atom C12A            has ADP max/min Ratio .....        3.1 oblate
PLAT230_ALERT_2_C Hirshfeld Test Diff for    S1A      --C10A     .        5.2 s.u.
```

Author Response: This can show possible disorder but the disordered model does not help in the modeling of this crystal.

```
PLAT230_ALERT_2_C Hirshfeld Test Diff for    S2A      --C8A      .         6.7 s.u.
```

Author Response: This can show possible disorder but the disordered model does not help in the modeling of this crystal.

```
PLAT230_ALERT_2_C Hirshfeld Test Diff for    S1D      --C10B     .         7.0 s.u.
```

Author Response: This can show possible disorder but the disordered model does not help in the modeling of this crystal.

```
PLAT230_ALERT_2_C Hirshfeld Test Diff for    S4D      --C11B     .         5.2 s.u.
```

Author Response: This can show possible disorder but the disordered model does not help in the modeling of this crystal.

```
PLAT234_ALERT_4_C Large Hirshfeld Difference S5A      --C15A     .        0.17 Ang.
PLAT234_ALERT_4_C Large Hirshfeld Difference S6A      --C14A     .        0.16 Ang.
PLAT234_ALERT_4_C Large Hirshfeld Difference C12A     --C13A     .        0.16 Ang.
PLAT234_ALERT_4_C Large Hirshfeld Difference N2B      --C7D      .        0.16 Ang.
PLAT241_ALERT_2_C High   'MainMol' Ueq as Compared to Neighbors of         S4A Check
PLAT241_ALERT_2_C High   'MainMol' Ueq as Compared to Neighbors of         S5C Check
PLAT241_ALERT_2_C High   'MainMol' Ueq as Compared to Neighbors of         S3B Check
PLAT241_ALERT_2_C High   'MainMol' Ueq as Compared to Neighbors of         S4D Check
PLAT906_ALERT_3_C Large K Value in the Analysis of Variance ......       8.984 Check
PLAT906_ALERT_3_C Large K Value in the Analysis of Variance ......       2.737 Check
PLAT971_ALERT_2_C Check Calcd Resid. Dens.  0.09Ang From S2C            1.53 eA-3
PLAT977_ALERT_2_C Check Negative Difference Density on H8D       .      -0.43 eA-3
PLAT977_ALERT_2_C Check Negative Difference Density on H14K      .      -0.31 eA-3
```

Alert level G

```
PLAT007_ALERT_5_G Number of Unrefined Donor-H Atoms .............            4 Report
PLAT072_ALERT_2_G SHELXL First  Parameter in WGHT  Unusually Large        0.20 Report
PLAT171_ALERT_4_G The CIF-Embedded .res File Contains EADP Records           2 Report
PLAT720_ALERT_4_G Number of Unusual/Non-Standard Labels ..........          16 Note
PLAT793_ALERT_4_G Model has Chirality at C5A         (Centro SPGR)           S Verify
PLAT793_ALERT_4_G Model has Chirality at C5B         (Centro SPGR)           R Verify
PLAT793_ALERT_4_G Model has Chirality at C6A         (Centro SPGR)           S Verify
PLAT793_ALERT_4_G Model has Chirality at C6B         (Centro SPGR)           R Verify
PLAT908_ALERT_2_G Max. Perc. Data with I > 2*s(I) per Res.Shell .       73.78% Note
PLAT910_ALERT_3_G Missing # of FCF Reflection(s) Below Theta(Min).           1 Note
PLAT912_ALERT_4_G Missing # of FCF Reflections Above STh/L=  0.600         969 Note
PLAT941_ALERT_3_G Average HKL Measurement Multiplicity ...........         2.8 Low
PLAT951_ALERT_5_G Calculated (ThMax) and CIF-Reported Kmax Differ            2 Units
PLAT957_ALERT_1_G Calculated (ThMax) and Actual (FCF) Kmax Differ            2 Units
PLAT978_ALERT_2_G Number C-C Bonds with Positive Residual Density.           0 Info
```

---

1 **ALERT level A** = Most likely a serious problem - resolve or explain
5 **ALERT level B** = A potentially serious problem, consider carefully
20 **ALERT level C** = Check. Ensure it is not caused by an omission or oversight
15 **ALERT level G** = General information/check it is not something unexpected

```
 2 ALERT type 1 CIF construction/syntax error, inconsistent or missing data
18 ALERT type 2 Indicator that the structure model may be wrong or deficient
 8 ALERT type 3 Indicator that the structure quality may be low
11 ALERT type 4 Improvement, methodology, query or suggestion
 2 ALERT type 5 Informative message, check
```

It is advisable to attempt to resolve as many as possible of the alerts in all categories. Often the minor alerts point to easily fixed oversights, errors and omissions in your CIF or refinement strategy, so attention to these fine details can be worthwhile. In order to resolve some of the more serious problems it may be necessary to carry out additional measurements or structure refinements. However, the purpose of your study may justify the reported deviations and the more serious of these should normally be commented upon in the discussion or experimental section of a paper or in the "special_details" fields of the CIF. checkCIF was carefully designed to identify outliers and unusual parameters, but every test has its limitations and alerts that are not important in a particular case may appear. Conversely, the absence of alerts does not guarantee there are no aspects of the results needing attention. It is up to the individual to critically assess their own results and, if necessary, seek expert advice.

Publication of your CIF in IUCr journals

A basic structural check has been run on your CIF. These basic checks will be run on all CIFs submitted for publication in IUCr journals (*Acta Crystallographica*, *Journal of Applied Crystallography*, *Journal of Synchrotron Radiation*); however, if you intend to submit to *Acta Crystallographica Section C* or *E* or *IUCrData*, you should make sure that full publication checks are run on the final version of your CIF prior to submission.

Publication of your CIF in other journals

Please refer to the *Notes for Authors* of the relevant journal for any special instructions relating to CIF submission.

Validation response form

Please find below a validation response form (VRF) that can be filled in and pasted into your CIF.

```
# start Validation Reply Form
_vrf_PLAT196_abf43
;
PROBLEM: No TEMP record and _measurement_temperature .NE.       293 Degree
RESPONSE: ...
;
# end Validation Reply Form
```

PLATON version of 12/09/2022; check.def file version of 09/08/2022

Datablock abf43 - ellipsoid plot

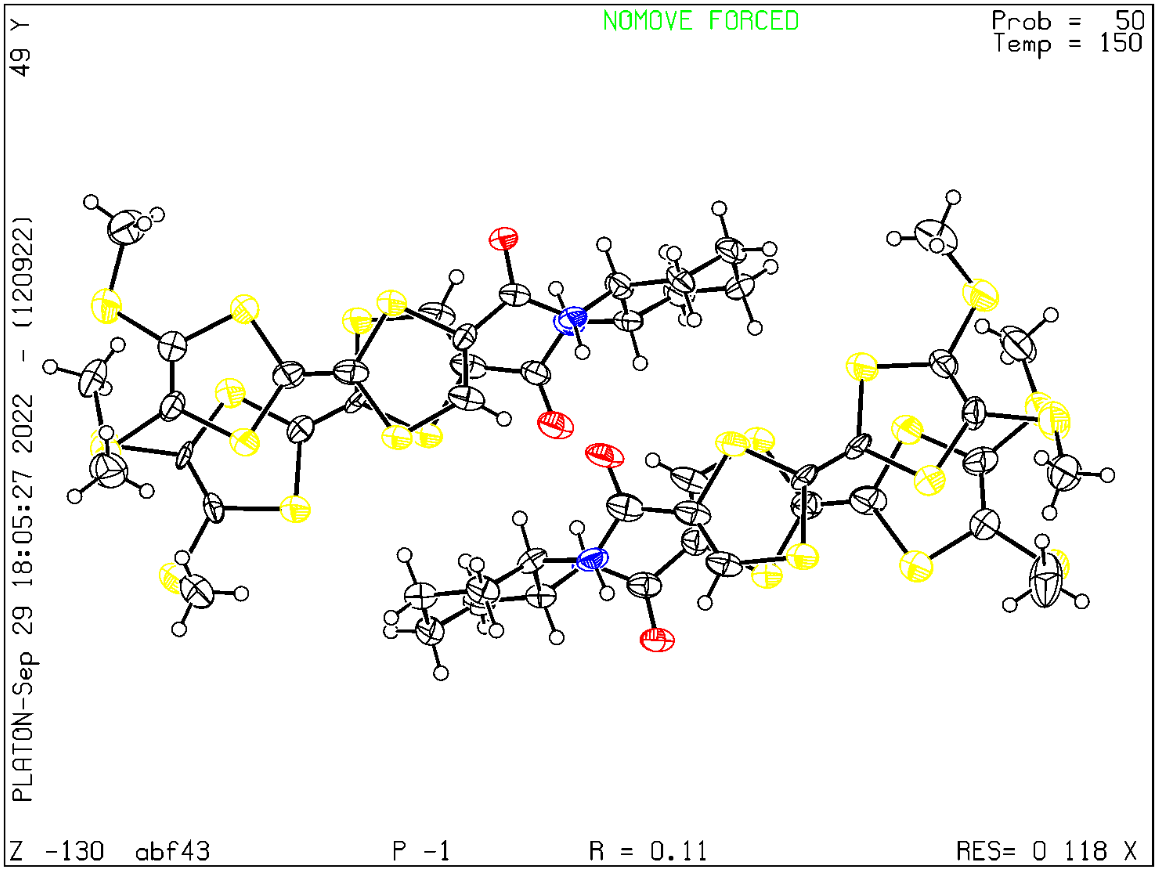

Supplement: Supplementary file 1 [file checkcif-diamide-revised.pdf › checkcif-diamide-revised.pdf]
